# Regulating Rac in the Nervous System: Molecular Function and Disease Implication of Rac GEFs and GAPs

**DOI:** 10.1155/2015/632450

**Published:** 2015-03-24

**Authors:** Yanyang Bai, Xiaoliang Xiang, Chunmei Liang, Lei Shi

**Affiliations:** JNU-HKUST Joint Laboratory for Neuroscience and Innovative Drug Research, Jinan University, Guangzhou, Guangdong 510632, China

## Abstract

Rho family GTPases, including RhoA, Rac1, and Cdc42 as the most studied members, are master regulators of actin cytoskeletal organization. Rho GTPases control various aspects of the nervous system and are associated with a number of neuropsychiatric and neurodegenerative diseases. The activity of Rho GTPases is controlled by two families of regulators, guanine nucleotide exchange factors (GEFs) as the activators and GTPase-activating proteins (GAPs) as the inhibitors. Through coordinated regulation by GEFs and GAPs, Rho GTPases act as converging signaling molecules that convey different upstream signals in the nervous system. So far, more than 70 members of either GEFs or GAPs of Rho GTPases have been identified in mammals, but only a small subset of them have well-known functions. Thus, characterization of important GEFs and GAPs in the nervous system is crucial for the understanding of spatiotemporal dynamics of Rho GTPase activity in different neuronal functions. In this review, we summarize the current understanding of GEFs and GAPs for Rac1, with emphasis on the molecular function and disease implication of these regulators in the nervous system.

## 1. Introduction

Rho family GTPases constitute a distinct family of guanine nucleotide-binding proteins which belongs to the superfamily of Ras-related GTPases. Rho GTPases are key regulators of the actin cytoskeletal dynamics, play crucial roles in various aspects of brain development, and are implicated in a number of neuropsychiatric and neurodegenerative diseases [1–5]. More than 20 mammalian members of Rho family GTPases have been described, including Rho-like (RhoA, RhoB, and RhoC), Rac-like (Rac1, Rac2, Rac3, and RhoG), Cdc42-like (Cdc42, TC10/RhoQ, TCL/RhoJ, Wrch1/RhoU, and Chp/Wrch2/RhoV), Rnd (Rnd1, Rnd2, and Rnd3/RhoE), Rho-BTB (Rho-BTB1 and RhoBTB2), RhoD, Rif/RhoF, and RhoH/TTF [6, 7]. Like all GTP-binding proteins, Rho GTPases contain sequence motifs for binding to GDP or GTP, thus acting as bimolecular switches, cycling between an inactive GDP-bound state and an active GTP-bound state. Activity of Rho GTPases is tightly controlled by the coordinated action of two classes of regulatory proteins: guanine nucleotide exchange factors (GEFs), which activate Rho GTPases by catalyzing the exchange of bound GDP for GTP, enabling them to recognize and activate downstream effectors, and GTPase-activating proteins (GAPs), which suppress Rho GTPases by enhancing their intrinsic rate of GTP hydrolysis to GDP.

Two types of GEFs for Rho GTPases have been identified. Dbl- (diffuse B-cell lymphoma-) like GEFs, the classical GEFs, are characterized by the presence of a DH (Dbl homology) domain followed by a PH (pleckstrin homology) domain [8]. The DH domain is known to be responsible for the catalytic exchange activity of Rho GEFs, whereas the PH domain regulates lipid binding and membrane targeting. Another type of GEFs is the Dock (dedicator of cytokinesis) family atypical GEFs, which contains a Dock homology region (DHR) 1-DHR2 module instead of the PH-DH module. DHR1-DHR2 module plays similar roles as PH-DH module, of which DHR1 is important for the phospholipid-binding and membrane targeting of Docks, and DHR2 is responsible for its GEF activity [9]. On the other hand, Rho GAPs are usually large multidomain proteins characterized by the presence of a conserved Rho GAP domain and various function domains [10]. A variety of GEFs and GAPs have been identified to govern the activity of Rho GTPases in neuronal development and to be associated with neurological diseases [5, 11–13].

Rac1 is one of the most well-studied Rho GTPases which controls a wide range of cellular events of neuronal morphogenesis and motility [14]. Rac1 is a master protein that directs actin polymerization and cytoskeletal changes through activating a series of signaling pathways, thus acting as a converging sensor molecule that conveys divergent upstream signals. To understand the spatiotemporally dynamic regulation of Rac1 activity in the nervous system, this review summarizes the current findings of more than 30 Rac GEFs (including both the Dbl-like and atypical GEFs) and GAPs (Table 1). Both the molecular functions and the disease relevance of these regulators in the nervous system will be discussed. Most of these Rac GEFs or GAPs also act on other Rho GTPases (such as RhoA or Cdc42) or even other subfamilies of Ras-related GTPases (such as Ras and Rab). For these regulators, we will emphasize their actions on Rac and will try to discuss how their actions on Rac or other GTPases are differentially regulated.

## 2. Dbl-Like Rac GEFs

### 2.1. Tiam

Tiam1 (T-lymphoma invasion and metastasis 1) is one of the most extensively studied Rac GEFs in nervous and other systems. Tiam1 is a well-known regulator for synapse formation and plasticity. Tiam1 interacts with both EphB receptors and the synaptic neurotransmitter receptors, N-methyl-D-aspartate (NMDA) receptors, at the postsynaptic sites [15, 16]. The Rac GEF activity of Tiam1 is upregulated by CaMKII (Ca^2+^/Calmodulin-dependent protein kinase II) dependent phosphorylation and EphB activation, leading to elevated Rac1 activity and spine formation [15, 16]. Moreover, Tiam1 forms a complex with Par3 (partitioning defective gene 3), a regulatory protein for cell polarization, and is restricted to the synapse. This regulation is important for the local activation of Rac1 and spine morphogenesis [17]. Interestingly, Tiam1 is also regulated by MAP1B (microtubule-associated protein 1B) and participates in NMDA-induced long-term depression (LTD) through Rac1-dependent endocytosis of AMPA (*α*-amino-3-hydroxy-5-methyl-4-isoxazolepropionic acid) receptors and spine shrinkage [18]. These seemingly contradictory findings of Tiam1 on both spine formation and elimination have been possibly explained by the following study. Tiam1 interacts and cooperates with BCR (breakpoint cluster region), a Rac GAP at the synapse, and the two counteract each other on Rac1 activity and spine morphogenesis [19]. This Rac GEF-GAP complex possibly secures a dynamic and balancing module for regulating spine structure, receptor internalization, and synaptic plasticity, whereas disrupting either component may cause abnormal activation of the other.

Besides regulating synaptic function, Tiam1 plays multifaceted roles in developmental stages of the nervous system. Tiam1 is highly expressed in the intermediate zone and the cortical plate of developing cortex. The Rac GEF activity of Tiam1 is required for the radial migration of newborn cortical neurons, and both Rac1-regulated leading process formation and JNK-regulated microtubule organization are implicated in Tiam1-regulated neuronal migration [20]. Moreover, Tiam1 acts as a converging transducer of a number of signals, including neurotrophins (such as nerve growth factor (NGF) and brain-derived neurotrophic factor (BDNF)), ephrins, and Wnts, to regulate neurite outgrowth and neural differentiation [21–24]. Tiam1 is also involved in the development of the myelinating glial cells oligodendrocytes and Schwann cells, suggesting a role of Tiam1 for the myelination of neurons in both central and peripheral nervous system [25, 26]. Moreover, the gene expression of Tiam1 is downregulated in specific neuronal types in response to cocaine and oxygen/glucose deprivation, which is one of possible molecular changes that cause synaptic structural or functional alterations in these pathological conditions [27, 28].

Tiam2, also known as STEF (Sif- and Tiam1-like exchange factor), is the second member of Tiam protein family. Tiam2 is far less studied than Tiam1, and its neuronal function revealed thus far is the regulation of neurite outgrowth [29, 30]. In particular, protein kinase A (PKA) dependent phosphorylation of Tiam2 activates the Rac GEF activity of Tiam2, which is a critical signaling pathway underlying dibutyryl cAMP (dbcAMP) induced neurite extension of neuroblastoma cells [30].

### 2.2. Trio and Kalirin

Trio and Kalirin (also known as Duo), both having several isoforms, are multidomain containing Rac GEFs that share high sequence homology. Trio is ubiquitously expressed and plays critical roles in early development of the nervous system, whereas Kalirin is specifically enriched in brain and elicits essential regulatory effects on synapse morphogenesis and function.

Mice with complete deletion of Trio display embryonic lethality with malformed myofibers and defective organizations in several brain regions including hippocampus and olfactory bulb [31]. Further characterization of these Trio knockout mice revealed that Trio transduces signaling from the chemoattractant netrin-1 through binding to the netrin-1 receptor DCC (deleted in colorectal cancer), thus guiding the outgrowth and projection of commissure axons of cortical neurons [32]. Trio is tyrosine phosphorylated by Fyn, a member of Src family kinases, in response to netrin-1 stimulation, which is essential for DCC localization and netrin-induced Rac1 activation and axon growth [33]. Moreover, Trio is implicated in NGF-mediated neurite outgrowth via binding to the membrane protein Kidins220/ARMS [34]. Several neuronal isoforms of Trio have been identified and they also exhibit activities on neurite outgrowth [35, 36]. To understand the neuronal roles of Trio, mice with specific deletion of Trio in the developing nervous system have been generated. These mice show remarkably reduced brain size and body weight, severe ataxia, and neonatal death. Disrupted cerebellum development including aberrant granule cell migration and abnormal neurite growth is observed in these mice, suggesting a critical role of Trio in cerebellum development [37].

Alternative splicing of the* KALIRIN* gene (*KALRN*) generates several transcripts encoding functionally distinct proteins, among which Kalirin-7 is the most prevalent isoform in mature neurons. Kalirin-7 is one of the critical integrators localized at the postsynaptic density of excitatory synapses to promote activity-dependent dendritic spine morphogenesis. Kalirin-7 is regulated at the synaptic areas through several mechanisms. First, Kalirin-7 is targeted to the synaptic membrane by interacting with synaptic proteins, such as PSD-95/PDZ-containing proteins and the N-cadherin adhesion molecule complex, leading to local activation of Rac1 and spine formation [38, 39]. Second, Kalirin-7 is extensively phosphorylated at the postsynaptic density, indicating that it is a converging signaling target and dynamically regulated by multiple kinases at the synapses [40]. In particular, the Rac GEF activity of Kalirin-7 is activated by synaptic EphB2 receptors and CaMKII via tyrosine and threonine phosphorylation, respectively [41, 42]. Moreover, Kalirin-7 is regulated by 5-HT_2A_ serotonin receptors and participates in serotonin-modulated spine size increase of cortical pyramidal neurons [43]. Besides regulating spine structure, Kalirin-7 also binds to the neurotransmitter NMDA and AMPA receptors and regulates their expressions and functions [42, 44]. To understand the* in vivo* roles of Kalirin-7 and other Kalirins in synapse structure and function, mice with only Kalirin-7 isoform deleted and mice with the* KALRN* gene deleted (in which all the Kalirin isoforms are absent) were generated. Interestingly, different phenotypical abnormalities were observed in these two lines of mice. For instance, spine morphogenesis and glutamatergic neurotransmission in cortex, but not hippocampus, are significantly reduced in* KALRN* knockout mice, and these mice exhibit age-dependent behavioral deficits such as reduced working memory, sociability, prepulse inhibition, and hyperactivated locomotion [45]. By contrast, only modest alterations are observed in the hippocampus, possibly associated with impaired fear conditioning in these mice [46]. On the other hand, Kalirin-7 knockout mice showed decreased spine density in hippocampus and abnormal anxiety-like behavior, but locomotion and spatial working memory are normal in these mice [47]. Since the expression of larger Kalirin isoforms, Kalirin-8, -9, and -12, is upregulated in Kalirin-7 knockout cortex [47], the discrepancies in these two lines of mice suggest nonoverlapping functions of Kalirin-7 and other Kalirins. The larger Kalirin isoforms have an additional RhoA GEF domain, which may thus contribute to their unique functions [48]. Indeed, Kalirin-9 and Kalirin-12 specifically regulate dendritic outgrowth and branching of cortical neurons, whereas overexpression of Kalirin-9 surprisingly decreases spine size and density [49, 50]. Kalirin-12 is also implicated in dynamin-dependent endocytosis in neuronal cells [51].

Being a critical synaptic regulator,* KALRN *has been found as a high risk gene of a variety of neurological diseases [52]. Both gene and protein expressions of Kalirin were decreased in the hippocampus of Alzheimer's disease (AD) patients in a study, and the lowered Kalirin level may contribute to higher iNOS (inducible nitric oxide synthase) activity observed in the hippocampal specimens of the patients [53, 54]. Moreover,* KALRN *expression is decreased in schizophrenia patients, and Kalirin-7 interacts with several schizophrenia-related proteins. The localization of Kalirin-7 and the duration of Rac1 activation are regulated by the schizophrenia-related factor DISC1 (disrupted-in-schizophrenia 1) [55]. Moreover, Kalirin-7 is a downstream mediator of the schizophrenia-related neuregulin/ErbB4 signaling, regulating dendritic spine morphogenesis of cortical pyramidal neurons and dendritic growth of cortical interneurons [56, 57]. Importantly, a sequence variant of* KALRN* found in schizophrenia patients encodes a Kalirin-7 mutant with diminished Rac GEF activity, and this mutant fails to increase spine density and size [58]. Expression of Kalirin-9, on the other hand, was found to be upregulated in schizophrenia subjects [49]. Moreover, Kalirin has been shown as a converging modulator in various pathological conditions such as those induced by cocaine and ischemia [59–61].

### 2.3. PIX

The PIX (p21-activated kinase (PAK) interacting exchange factor) protein family includes *α*PIX (ARHGEF6) and *β*PIX (ARHGEF7). * αPIX/ARHGEF6* gene is one of the causative genes of X-linked intellectual disability (ID) [62]. *α*PIX is expressed primarily in the hippocampus and is localized to the postsynaptic density of excitatory neurons [63, 64]. *α*PIX regulates spine morphogenesis through interacting with the postsynaptic adaptor protein GIT1 (G-protein coupled receptor kinase-interacting protein 1) and activation of Rac and PAK3 [63, 65]. In the early development of neurons, *α*PIX promotes axon and dendrite branching and participates in dendritic Golgi translocation in response to reelin [66, 67]. *α*PIX knockout mice exhibit disrupted synaptic plasticity and a series of behavioral abnormalities, including impaired spatial and complex learning and less behavioral control in mildly stressful situations, resembling typical ID symptoms [64].

*β*PIX plays roles at both excitatory and inhibitory synapses. At excitatory synapse, *β*PIX forms a complex with important postsynaptic molecules including Shank and GIT1 and regulates synaptogenesis [68]. *β*PIX is phosphorylated by CaMKII in response to neuronal activity induced Ca^2+^ influx, which causes activation of *β*PIX toward Rac1 and spine formation [69]. *β*PIX also forms a complex with cadherin, *β*-catenin, and scribble at presynaptic sites and regulates synaptic vesicle positioning [70]. Interestingly, the GIT1 and *β*PIX complex is also localized to inhibitory synapses and regulates the synaptic stability of GABA_A_ receptors [71]. The ability of *β*PIX in regulating both excitatory and inhibitory synapses suggests that it may be an essential modulator of synaptic balance. Moreover, *β*PIX is involved in several signaling pathways that regulate neurite and dendritic outgrowth [72–74]. This is controversial to a recent report that *β*PIX knockdown has no effect on dendritic growth and branching [66]. These observations may be due to the presence of different isoforms of *β*PIX that may play additional functions during neuronal development [66, 75].

### 2.4. Farp

Farp (4.1, ezrin, radixin and moesin (FERM), RhoGEF (ARHGEF), and pleckstrin domain protein) family includes two closely related members, Farp1 and Farp2. Both Farps function as mediators of Semaphorin (Sema)/Plexin signaling. Farp1 interacts with PlexinA1 receptors and is required for Sema3A-promoted dendritic arborization of hippocampal neurons, which is a neuronal activity-dependent process [76]. Moreover, Farp1 is a responsive gene of retinoids in the developing spinal cord, where it mediates Sema6A/PlexinA4 signaling induced dendritic growth of spinal motoneurons [77]. On the other hand, although Farp2 also binds to PlexinA1 receptors, this interaction is diminished by Sema3A during axonal repulsion of dorsal root ganglion (DRG) neurons [78]. Dissociation of Farp2 from PlexinA1 increases Farp2's GEF activity toward Rac1 and subsequently activates other signaling events, leading to repulsion and decreased adhesion of axons [78]. A recent study dissected the functional roles of different cytoplasmic domains of PlexinA4 and compared the requirement of Farp1 and Farp2 in Sema3A/PlexinA4 signaling. It reveals that Farp1 and Farp2 bind to PlexinA4 in different fashions, and only Farp2 is required for Sema3A/PlexinA4 induced growth cone collapse of DRG neurons and dendritic growth of cortical neurons [79]. Besides acting as an effector of Sema/Plexin signals, it is found that Farp1 interacts with SynCAM1 (synaptic cell adhesion molecule 1), a synaptogenic protein, and regulates synapse formation. Farp1 works together with SynCAM1 to promote spine morphogenesis through activation of Rac1 and increase of F-actin polymerization in spine heads. Farp1 and SynCAM1 also activate a retrograde signaling on the modulation of presynaptic active zones [80].

### 2.5. P-Rex

P-Rex (phosphatidylinositol (3,4,5)-trisphosphate-dependent Rac exchanger) family, including P-Rex1 and P-Rex2, is activated by both PI3K (phosphoinositide 3-kinase) and GPCRs (G-protein coupled receptors). P-Rex1 is localized to the distal tips of neurites and axonal growth cones and regulates NGF-stimulated neurite outgrowth through activating Rac1 and Rac3 [81]. Moreover, P-Rex1 is expressed in the developing cortex and regulates both radial migration and tangential migration of newborn pyramidal neurons under the control of neurotrophins (such as NGF and BDNF) and ephrin-B1, respectively [82, 83].

Expression of P-Rex2 in the brain is much more limited and is most prominently expressed in cerebellar Purkinje cells. Aberrant dendrite morphology of Purkinje cells was observed in P-Rex2 knockout mice, associated with motor coordination deficits. P-Rex1 and P-Rex2 double knockout mice exhibit more severe motor coordination defects together with ataxia, abnormal posture, and gait [84]. This suggests that P-Rex1 and P-Rex2 cooperatively regulate cerebellum function. Further studies using P-Rex1/2 double knockout mice show that PI3K-P-Rex signaling is important for late phase long-term potentiation (LTP) at the parallel fiber-Purkinje cell synapse in cerebellum [85].

### 2.6. Vav

Three mammalian Vavs, Vav1, Vav2, and Vav3, have been identified in Vav protein family. Vav1 is preferentially expressed in immune system, whereas Vav2 and Vav3 are more ubiquitous with higher expression in developing brain. A number of studies have revealed that Vav2 and Vav3, in particular Vav2, participate in diverse signaling pathways to regulate neurite outgrowth and branching* in vitro* and in* Xenopus* spinal neurons [86–88]. Investigation of Vav2 and Vav3 single or double knockout mice has provided more information about the functional roles of these proteins in the nervous system. In early developmental stages, Vav2 and Vav3 are found to mediate ephrin/Eph signaling regulated axon guidance of ipsilateral retinogeniculate projections [89]. Vav2 binds to activated EphA4 receptors and its Rac GEF activity was stimulated by ephrin-A1. Such regulation induces endocytosis of the ephrin/Eph complex and a subsequent growth cone collapse of cultured retinal ganglion cells (RGCs). Importantly, deletion of both Vav2 and Vav3 in mice results in abnormal axon projection of RGCs to the dorsal lateral geniculate nucleus. In later developmental stages, Vav2 and Vav3 interact with TrkB receptors and are transiently activated upon TrkB stimulation by BDNF, leading to the activation of Rac1 [90]. Vav2 and Vav3 are dispensable for basal dendritic spine formation but are required for BDNF-induced rapid spine enlargement and theta-burst-stimulated LTP in hippocampus [90]. Other abnormalities identified in Vav2/Vav3 double knockout mice include delayed degeneration, revascularization and regeneration of peripheral nerves, and optic neuropathy associated with ocular deficits that are highly resemblance of glaucoma-like phenotypes [91, 92].

To address whether individual Vav plays nonoverlapping biological roles, single knockout mice of each Vav have been generated. In particular, two additional functions of Vav3 in the nervous system have been found. First, Vav3, but not Vav2, contributes to the development of cerebellum [93]. Vav3 regulates the precise timing of several developmental processes at postnatal stages, including dendritogenesis of Purkinje cell and the survival and migration of the granule cells. In line with the morphological defects of cerebellum, Vav3 knockout mice exhibit abnormalities in cerebellum-related behaviors such as motor coordination and gaiting patterns [93]. Second, Vav3 specifically regulates the axon guidance of a subset of GABAergic neurons in the ventrolateral medulla (VLM), a brainstem area. This regulation controls the precise GABAergic transmission in VLM, which is eventually important for the modulation of blood pressure and respiratory rates [94]. Moreover, it was recently found that* VAV3* is a candidate gene for schizophrenia, pointing to the importance of investigating Vav3 in the molecular pathophysiology of schizophrenia [95].

### 2.7. Plekhg4

Plekhg4, a GEF enriched in adult cerebellum, has been identified to be linked with autosomal dominant spinocerebellar ataxia, a heritable neurodegenerative disease [96]. When expressed heterologously in fibroblast cells, Plekhg4 possesses general GEF activities toward RhoA, Rac1, and Cdc42 and is capable of inducing actin-dependent formation of lamellipodia and filopodia [97]. The stability and subcellular localization of Plekhg4 can be regulated by the chaperon complex of heat shock proteins [97]. Nonetheless, the function of Plekhg4 in the nervous system, especially the cerebellum, and how its dysfunction leads to cerebellar impairments still await further characterization.

### 2.8. GEFT

GEFT, a small GEF that contains primarily a DH and a PH domain, is widely expressed in various brain regions. Overexpressed GEFT promotes dendrite and spine growth in hippocampal neurons and facilitates retinoic acid and dbcAMP induced neurite outgrowth in neuroblastoma cells [98, 99]. Although GEFT may activate both Rac1 and Cdc42, its effect on neurite growth is possibly via Rac1/PAK pathway [98].

### 2.9. RasGRF

RasGRF protein family, including RasGRF1 and RasGRF2, has dual GEF activities toward both Ras and Rac and is expressed predominantly in mature neurons. RasGRFs play multiple roles in neurite outgrowth, synaptic plasticity, and neuronal behaviors [100, 101]. In particular, RasGRF1 exhibits differential roles in regulating LTD, LTP, and dentate gyrus neurogenesis in an age-dependent manner [102, 103]. RasGRF1 directly couples to NMDA receptor 2B subunits (NR2B) to participate in the induction of LTD and dendrite complexity development [102, 104, 105]. On the other hand, association of RasGRF2 with NMDA receptor 2A subunits (NR2A) is required for LTP generation [102, 106]. As critical mediators of synaptic function, both RasGRFs are found to be essentially involved in physiological and pathological neural behaviors. For instance, RasGRF1 regulates learning and memory and is implicated in epilepsy, striatum-dependent motor behavioral deficits induced by cocaine, L-dopa, or amphetamine [107–112]. RasGRF2 is important for contextual discrimination and is found to be a responsible molecule for alcohol-induced reinforcement [113, 114].

Given that RasGRFs activate both Ras and Rac1, detailed analysis has been performed to provide further information about whether both small GTPases or only one of them is important for specific RasGRF's function. In particular, Rac1 signaling is differentially activated in RasGRF1-mediated LTD [102]. Similarly, although the Ras GEF activity of RasGRF2 regulates synaptic strength and NMDA current, only its Rac GEF activity is activated immediately following stimulation of NMDA, which is in turn required for the rapid spine enlargement and LTP generation [115]. Importantly, the Rac GEF activity of RasGRFs may be differentially regulated by protein interaction and phosphorylation. For instance, the Rac GEF activity of RasGRF1 is specifically inhibited by the microtubule-destabilizing factor SCLIP (SCG10-like protein), which antagonizes the neurite outgrowth induced by RasGRF1 [116]. Moreover, the Rac GEF activity of RasGRF2 is decreased by p35/Cdk5 via phosphorylation [117].

### 2.10. Alsin

The gene encoding Alsin,* ALS2*, is a causative gene of several motoneuron degenerative diseases, including juvenile amyotrophic lateral sclerosis (ALS), primary lateral sclerosis, and infantile-onset ascending hereditary spastic paralysis [118]. Alsin has dual GEF activities for both Rab5, a member of Rab GTPases essential for protein trafficking through early stages of the endocytic pathway, and Rac1. A battery of studies in cells and in* ALS2* knockout mice have revealed a broad range of cellular functions of Alsin, including protection of motoneuron survival, endosomal trafficking of neuronal membrane proteins, and neurite outgrowth [119–124]. Both Rab5 and Rac1 contribute to these functions mediated by Alsin. In particular, knockdown of Alsin in cultured rat spinal motoneurons leads to reduced endosome size, abnormal protein trafficking, and elevated neuronal death [121]. Activation of Rac1, but not Rab5, rescues these defects in Alsin deficient neurons [121]. Thus, although Rab5 may contribute to Alsin-regulated endosomal trafficking, Rab5 is not essentially required in Alsin-regulated neuronal functions. In agreement with this finding, the membrane and endosomal localization of Alsin is reciprocally regulated by activated Rac1, suggesting that activation of Rac1 may be a signaling event prior to that of Rab5 [125, 126]. Moreover, the Rac GEF activity, in particular Rac1/PI3K/Akt3 pathway, is involved in Alsin-regulated protective effects against motoneuron death induced by mutant forms of* SOD1* (superoxide dismutase 1), another causative gene in ALS [120, 127]. Rac1/PAK pathway is also important for Alsin-regulated neurite outgrowth [119]. However, Alsin knockout mice exhibit no obvious motoneuron death and only mild motor defects [118]. The poor recapitulation to phenotypes in human diseases may be due to compensatory effects from other ALS-related genes such as* SOD1*. Indeed, loss of* ALS2 *in a ALS-related* SOD1* mutant mice exacerbates neurotoxicity, accumulation of misfolded proteins, and motor dysfunction of the mutant mice [128].

## 3. Dock Family Atypical Rac GEFs

Dock (dedicator of cytokinesis) protein family is a family of atypical GEFs which contains 11 members, of which Dock1–5 activate Rac1 and Dock6 and Dock7 activate both Rac1 and Cdc42 [129]. Except Dock5, the other six members of these Rac GEF activity-possessing Docks all have known functions in the nervous system. Dock1, also called Dock180, is involved in the regulation of axon guidance and dendritic spine morphogenesis. Dock180 binds to the netrin receptor DCC and mediates netrin-induced Rac1 activation and axon growth [130]. Interestingly, Dock180 is also important for the axon pruning induced by ephrin-B3 reverse signaling and RhoG, suggesting a bifaced role of Dock180 in axon attraction and repulsion [131, 132]. Moreover, Dock180 promotes Rac1 activation and leads to dendritic spine morphogenesis in hippocampal neurons [133].

Dock2 is expressed exclusively in microglia and is implicated in neuroinflammation of AD pathology. It has been shown that the number of Dock2-expressing microglia is abnormally increased in brains of AD patients and the expression of Dock2 is positively regulated by prostaglandin E2 receptors [134]. Additionally, Dock2 deficiency significantly reduces the area and size of *β*-amyloid (A*β*) plaque in cerebral cortex and hippocampus of a mouse model of AD [135].

Dock3 plays major roles in neurite outgrowth and neuroprotection and is implicated in several neurological diseases such as AD and glaucoma [136]. Dock3 mediates several molecular events that are important for BDNF-induced neurite and axon growth [137, 138]. More importantly, Dock3 exerts several neuroprotective effects, whereas loss of Dock3 leads to axon degeneration [139]. Dock3 decreases the secretion of APP (amyloid precursor protein) and A*β* peptide by accelerating the proteasome-dependent degradation of APP [140]. Moreover, Dock3 ameliorates the neurotoxicity induced by NMDA receptors via interacting with the C-terminus of NMDA receptor subunits [141, 142]. These findings suggest that Dock3 is a potential therapeutic target for nerve injury and degeneration. Indeed, two recent findings have shown that Dock3 stimulates neuroprotection after optic nerve injury and protects myelin in a demyelination model of multiple sclerosis [143, 144].

The gene encoding Dock4 has been found to be associated with several neuropsychiatric diseases, including autism, dyslexia, and schizophrenia [145–147]. Dock4 regulates neurite and dendrite outgrowth through Rac1-dependent actin cytoskeleton reorganization [148]. Dock4 is also important for spine morphogenesis of hippocampal neurons [149].

Dock6 has been found to promote neurite outgrowth and regulate axonal growth and regeneration of sensory neurons [150, 151]. Although Dock6 is capable of activating both Rac1 and Cdc42* in vitro*, it preferentially activates Rac1 in DRG neurons [151]. The GEF activity of Dock6 towards Rac1 is regulated by phosphorylation/dephosphorylation at Ser1194 by Akt kinase and protein phosphatase PP2A.

Dock7 is highly expressed in the developing brain and has been found to play important roles in several neuronal developmental processes. Dock7 regulates the neurogenesis in the neocortex by promoting the differentiation of radial glial progenitor cells into basal progenitors and neurons [152]. Dock7 also plays a role in controlling neuronal polarity and axon formation [153]. A recent study has revealed that Dock7 is expressed in chandelier cells, an important type of interneurons for modulating cortical circuits, and regulates axonal terminal development of these cells under the control of ErbB4 receptors [154]. Notably, mutations in* DOCK7 *were found in individuals with epileptic encephalopathy and cortical blindness [155]. In the peripheral nervous system, Dock7 is important for the development of Schwann cells, the glial cells that ensheath the axons of motor and sensory neurons. Dock7 negatively regulates the differentiation of Schwann cells and the onset of myelination in both primary Schwann cells* in vitro* and sciatic nerves* in vivo* [156]. Moreover, Dock7 promotes Schwann cell migration mediated by neuregulin-ErbB2 receptors [157].

## 4. Rac GAPs

### 4.1. BCR and ABR

BCR and ABR (active BCR-related) are two closely related Rac GAPs expressed mainly in the brain. ABR and BCR have both GAP and GEF domains but only exhibit GAP activity* in vivo*. Knockout of both ABR and BCR or either of them in mice leads to elevated Rac1 activity in the brain [158, 159]. Defects observed in the knockout mice include functional and structural abnormalities of astroglia in postnatal cerebellar development, increased dendritic arborization and spine number, and defective LTP maintenance in the hippocampus [158–160]. Moreover, BCR coexists with Tiam1 as a Rac GEF-GAP complex to regulate spine morphogenesis and synaptic plasticity under the control of BDNF-TrkB signals [19]. Either ABR or BCR knockout mice exhibit impaired spatial and object recognition memory [158]. The GAP activity of BCR can be promoted by phosphorylation at its Tyr^177^ residue [160]. Fyn and protein tyrosine phosphatase receptor T (PTPRT) are the upstream kinase and phosphatase, respectively, that regulate BCR activity via targeting the Tyr^177^ residue [160].

### 4.2. Chimaerin

Chimaerins are a family of Rac GAPs which contain a GAP domain homologous to that of BCR. Two subfamilies of chimaerins, *α*-chimaerins encoded by* CHN1* gene and *β*-chimaerins encoded by* CHN2* gene, are identified in mammals. There are three alternative spliced products of each of* CHN1* and* CHN2*: *α*1- and *β*1-chimaerins contain a C1 domain and a GAP domain, *α*2- and *β*2-chimaerin possess an additional SH2 domain at the N-terminus, and *α*3- and *β*3-chimaerin only contain a GAP domain [161].

Among the different members of chimaerins, *α*2-chimaerins are the most studied and have been revealed to play crucial functions in the nervous system. Mutation of the* CHN1* gene is one of the causes of Duane's retraction syndrome (DRS), a complex congenital eye movement disorder caused by aberrant axonal innervation of the extraocular muscle [162]. A number of heterozygous missense mutations in* CHN1* have been found in DRS and all cause hyperactivation, that is, gain of function, of the GAP activity of chimaerin and misguidance of oculomotor nerves [162]. Further molecular dissection has shown that *α*2-chimaerin acts as a downstream mediator of both the repellent signals of oculomotor axon guidance, that is, Sema3/PlexinA, and the attractant signals, that is, the chemokine CXCL12 and hepatocyte growth factor (HGF) [163]. The ability of *α*2-chimaerin to respond to both positive and negative signals suggests that *α*2-chimaerin represents a balancing intermediate which maintains the high sensibility of axons to the surrounding microenvironment.

Besides regulating axon guidance in oculomotor system, *α*2-chimaerin is also critical in the axon pathfinding of corticospinal tract and spinal cord neurons during motor circuit assembly. *α*2-Chimaerin functions as an indispensable effector of ephrin/Eph repellent signaling pathway to restrict the axons to project into the ipsilateral side of the spinal cord without crossing the midline [164–167]. Such function of *α*2-chimaerin controls alternate body movement, whereas knockout of *α*2-chimaerin results in locomotion defects and involuntary synchronous arrhythmic stepping, known as a rabbit-like hopping gait [164, 166–168]. The molecular regulation of *α*2-chimaerin activation includes membrane recruitment and tyrosine phosphorylation of *α*2-chimaerin by Eph receptors and binding to the adaptor protein Nck family, which leads to Rac1 in activation and growth cone collapse [164, 165, 169].

Interestingly, despite the hopping gait behavior, *α*2-chimaerin null mice exhibit enhanced contextual fear learning. Such behavior abnormalities are only observed when *α*2-chimaerin is genetically deleted in early developmental stages, but not in adulthood [170]. One of the roles of *α*2-chimaerin in early development is the regulation of the radial migration and positioning of newborn pyramidal neurons during corticogenesis. Such regulation is a key determinant of normal cortical excitability and seizure threshold in adulthood [171]. On the other hand, *α*1-chimaerin, the shorter splicing variant of *α*2-chimaerin, is enriched in later developmental stages and is not involved in gait behavior or contextual fear learning [170]. *α*1-Chimaerin plays a role in pruning of dendritic branches and spines, and such effect may be regulated by synaptic activity and interaction with NMDA receptors [172, 173].

The function of *β*-chimaerins in the nervous system is less understood comparing to *α*-chimaerins. One report has revealed that *β*2-chimaerin acts as a mediator of Sema3F signaling in regulating axonal pruning in developing hippocampus [174].

### 4.3. srGAP

The Slit-Robo GAP (srGAP) family is an F-BAR (Bin, Amphiphysin, and Rvs) domain containing GAP family that has four members, srGAP1, srGAP2, srGAP3, and ArhGAP4. Among these members, srGAP1 preferentially inhibits Cdc42, whereas srGAP2 and srGAP3 downregulate Rac1. ArhGAP4 may be implicated in the inhibition of axon growth, but its function has been largely unknown [175].

srGAP2 negatively regulates radial migration of cortical neurons through its F-BAR domain mediated increase of leading process branching [176]. The GAP activity of srGAP2 toward Rac1 partially contributes to srGAP2-induced neurite branching and migration inhibition [176]. Moreover, srGAP2 promotes maturation of dendritic spines but decreases sine density in the neocortex [177]. Notably, it is found that* SRGAP2* is one of the human-specific duplicated genes, which undergoes incomplete duplications that generate several partial* SRGAP2* products in the human brain [177, 178]. srGAP2C, encoded by one of the major duplications, forms dimers with srGAP2 and inhibits srGAP2 function on migrating inhibition and spine maturation, suggesting that srGAP2 is modulated by its paralogs in human brain [177].

srGAP3, also called MEGAP (mental disorder associated GAP protein) or WRP (WAVE (Wiskott-Aldrich syndrome protein verprolin-homologous) associated Rac GTPase-activating protein), is a factor linked to mental retardation [179]. srGAP3 controls the early stages of dendritic spine formation in an F-BAR domain dependent manner and is dispensable for the maintenance of spine density [180]. The* in vivo* functions of srGAP3 on synapse structure and function have been revealed in several lines of srGAP3 knockout mice. Mice with deletion of srGAP3 in brain show deficits in multiple long-term learning and memory tasks, including novel object recognition, water maze, and passive avoidance [180]. In another study, a line of complete srGAP3 knockout mice show schizophrenia-like behaviors, such as impaired social behavior, working memory and prepulse inhibition, more spontaneous tics, and exacerbated methylphenidate-induced locomotor hyperactivation [181]. However, the long-term memory is surprisingly normal in these complete srGAP3 knockout mice. Moreover, srGAP3 interacts with the actin regulatory scaffold WAVE-1 and regulates synapse morphogenesis and function. Mice with disrupted WAVE-1-srGAP3 interaction show decreased spine density, elevated LTP, and impaired retention of spatial memory [182]. Notably, srGAP3 knockout mice exhibit perinatal-onset hydrocephalus, which is possibly due to cerebral aqueductal occlusion caused by abnormal migration and differentiation of progenitor cells from the ventricular region into the corpus callosum [181, 183]. Studies in neuroblastoma cells and cultured embryonic neural progenitor cells have confirmed that srGAP3 negatively regulates neuronal differentiation and neurite outgrowth [184, 185]. Such effect of srGAP3 may involve interaction with Brg1 (Brahma-related gene 1), a modulator of chromatin remodeling enzymes, and an interplay of other srGAPs such as srGAP2 [186, 187]. Other functions of srGAP3 include the positioning of commissural axons of spinal cord neurons, possibly under the regulation of Slit-Robo signals [188].

### 4.4. p250GAP

p250GAP, also called RICS or Grit, shows GAP activity toward inhibition of RhoA, Rac1, and Cdc42.* p250GAP* gene was recently found as a candidate gene for susceptibility to schizophrenia, suggesting its potential importance in brain development and function [189]. p250GAP is a target of microRNA 132 (miR132), which downregulates p250GAP expression and upregulates Rac1 activity in a manner dependent on neuronal activity. This miR132-p250GAP pathway is required for dendrite development, the hormonal regulator leptin-induced synaptogenesis, and BDNF-induced axon branching of RGCs during retinocollicular/tectal map formation [190–192]. On the other hand, p250GAP interacts with and is recruited to spines by NMDA receptors, leading to a modulation of RhoA activity and spine morphology [193, 194]. Moreover, functions of p250GAP dependent on RhoA or Cdc42 have been revealed in regulating neurite/axon growth and neuronal migration of cortical neurons [195–197]. PX-RICS, a major isoform of p250GAP identified in the nervous system, also regulates neurite extension [198].

### 4.5. Rich

Rich (Rho GAP interacting with Cdc42-interacting protein 4 homologues) protein family contains two members, Rich1 and Rich2. Rich1, also called Nadrin (neuron-associated developmentally regulated protein), shows GAP activity toward RhoA, Cdc42, or Rac1. In particular, Rich1 interacts with PACSIN (protein kinase C and casein kinase 2 substrate in neurons), a neuronal adaptor protein, to regulate dendritic spine morphogenesis through modulating Rac1 activity [199]. Different splicing variants of Rich1 have been identified in neurons and some show inhibitory effects on NGF-induced neurite outgrowth [200].

Rich2 is a novel interacting protein of Shank3, a critical postsynaptic scaffolding protein. During LTP, Rich2-Shank3 interaction is increased in dendritic spines and the complex participates in exocytosis of AMPA receptor subunits through endosomal recycling [201]. Furthermore, Rich2 specifically inactivates Rac1 in neurons and regulates dendritic spine morphogenesis [202].

### 4.6. SH3BP1

An RNAi screening identified SH3BP1 (SH3-domain binding protein 1) as a downstream mediator of Sema3E/PlexinD1 signaling [203]. SH3BP1 binds to PlexinD1 receptors at resting state and is released from PlexinD1 complex upon Sema3E stimulation, which in turn leads to inactivation of Rac1 and cell collapse [203]. SH3BP1 is also implicated in Sema3A-mediated growth cone collapse [204]. Moreover, an SH3BP1 splice-variant BARGIN (BGIN), which possesses a C-terminus polyubiquitin (Ub) binding module, inactivates Rac1 in a poly-Ub-interaction dependent manner. BGIN-mediated Rac1 inhibition contributes to diminishing reactive oxygen species (ROS) in APP-related pathology of AD [205].

### 4.7. CrGAP/Vilse

CrossGAP (crGAP), also called Vilse, was identified as a Rac GAP that mediates Slit-Robo signaling in axon repulsion in* Drosophila* [206, 207]. In mammals, it was recently found that crGAP/Vilse interacts with CNK2 (connector enhancer of KSR-2), a scaffold protein implicated in ID, and regulates spine morphogenesis of hippocampal neurons [208].

### 4.8. MgcRacGAP

MgcRacGAP is a Rac GAP implicated in cytokineses. MgcRacGAP interacts with kinesin-6, a microtubule-based motor protein, and regulates F-actin distribution and movement of migrating cortical neurons [209].

## Figures and Tables

**Table 1:** Summary of Rac GEFs and GAPs in the nervous system.

Name (aliases)	Functions in the nervous system	Upstream signals	Neurological disease relevance	Other targeted GTPases
Dbl-like Rac GEFs				

Tiam1	Spine morphogenesis; neuronal migration; neurite outgrowth; neural differentiation; glial cell myelination	BDNF/TrkB; NGF/TrkA; NT3/TrkC; ephrinB1/EphB2; Wnts; CaMKII		Cdc42

Tiam2 (STEF)	Neurite outgrowth	dbcAMP		Cdc42

Trio	Axon guidance; neurite outgrowth; cerebellum development; neuronal cluster organization in hindbrain	Netrin/DCC; NGF; Notch		RhoA

Kalirin-7	Spine morphogenesis; synaptic plasticity; learning and memory; dendritic growth of interneurons; regulation of iNOS	EphB2; CaMKII; N-cadherin; 5-HT_2A_ serotonin receptors; neuregulin/ErbB4	Alzheimer's disease; ischemic stroke; schizophrenia; cocaine addiction; attention deficit hyperactivity disorder; Huntington's disease	

Kalirin-9	Dendritic outgrowth and branching; spine morphogenesis		Schizophrenia	RhoA

Kalirin-12	Dendritic outgrowth and branching; endocytosis			RhoA

*α*-PIX (ARHGEF6/Cool-2)	Spine morphogenesis; axon and dendrite branching; learning and memory	Reelin	X-linked mental retardation	Cdc42

*β*-Pix (ARHGEF7/Cool-1)	Spine formation; presynaptic vesicle positioning; GABA_A_ receptor stabilization; neurite and dendrite outgrowth	CaMKII		Cdc42

Farp1 (CDEP)	Dendritic arborization; spine morphogenesis; presynaptic active zone modulation	Sema3A/PlexinA1; Sema6A/PlexinA4; retinoids; SynCAM1		

Farp2 (FIR/FRG)	Axon guidance; dendrite growth	Sema3A/PlexinA1, PlexinA4		Cdc42

P-Rex1	Neurite outgrowth; neuronal migration; cerebellum development and function	NGF; BDNF; ephrin-B1		

P-Rex2	Cerebellum development and function	PI3K		

Vav2	Neurite outgrowth; axon guidance; spine development	Ephrin/Eph; BDNF/TrkB; NGF/PI3K		Cdc42

Vav3	Neurite outgrowth; axon guidance; spine development; cerebellum development; GABAergic neuron transmission in brainstem	Ephrin/Eph; BDNF/TrkB; NGF/PI3K	Schizophrenia	Cdc42

Plekhg4 (puratrophin-1)	Cerebellar function		Spinocerebellar ataxia	RhoA; Cdc42

GEFT	Neurite and dendrite growth	Retinoic acid; dbcAMP		Cdc42

RasGRF1	Neurite growth; synaptic plasticity; learning and memory; striatal function		Epilepsy; L-dopa-induced dyskinesia	Ras

RasGRF2	Neurite growth; synaptic plasticity; alcohol-induced reinforcement		Alcoholism	Ras

Alsin	Motoneuron protection; endosomal trafficking; neurite outgrowth		Amyotrophic lateral sclerosis; primary lateral sclerosis; infantile-onset ascending hereditary spastic paralysis	Rab5

Atypical Rac GEFs				

Dock1 (Dock180)	Spine morphogenesis	Netrin/DCC; RhoG		

Dock2	Microglia function and A*β* deposition	Prostaglandin E2	Alzheimer's disease	

Dock3 (MOCA/PBP)	Axonal outgrowth; neuroprotection	BDNF/TrkB	Alzheimer's disease; glaucoma; attention deficit hyperactivity disorder	

Dock4	Neurite and dendrite development; spine morphogenesis	Retinoic acid	Autism; dyslexia; schizophrenia	

Dock6 (Zir1)	Axonal growth and regeneration			Cdc42

Dock7 (Zir2)	Neuronal polarity; cortical neurogenesis; Schwann cell differentiation and myelination; axon terminal development of chandelier cells	Neuregulin/ErbB2, ErbB4	Epileptic encephalopathy and cortical blindness	Cdc42

Rac GAPs				

BCR	Dendrite growth; spine morphogenesis; astroglia development; learning and memory	BDNF/TrkB		Cdc42

ABR	Dendrite growth; spine morphogenesis; astroglia development; learning and memory			Cdc42

*α*1-Chimaerin (n-chimaerin)	Dendrite and spine development	Diacylglycerol	Duane's retraction syndrome	

*α*2-Chimaerin	Axon guidance in oculomotor and motor system; neuronal migration; cognitive function	Ephrin/Eph; Sema3/PlexinA; BDNF/TrkB; CXCL12; HGF	Duane's retraction syndrome	

*β*2-Chimaerin	Axonal pruning	Sema3F		

srGAP2 (FNBP2)	Neuronal migration; spine development; neurite outgrowth	Slit/Robo; valproic acid	Schizophrenia; epilepsy	Cdc42

srGAP3 (MEGAP/WRP)	Spine development; synaptic plasticity; learning and memory; neural progenitor cell differentiation and migration; neurite outgrowth	Slit/Robo; valproic acid	Hydrocephalus; X-specific mental retardation; schizophrenia; epilepsy	

ArhGAP4	Axon growth			

p250GAP (Grit/RICS/p250GAP/p200RhoGAP/GC-GAP)	Dendritic and spine morphogenesis; axon guidance and branching; neurite outgrowth; neuronal migration	BDNF; NGF/TrkA; leptin	Schizophrenia	RhoA; Cdc42

PX-RICS	Neurite outgrowth			

Rich1 (Nadrin)	Spine morphogenesis; neurite outgrowth; astrocyte differentiation	NGF		RhoA; Cdc42

Rich2	Spine morphogenesis and synaptic plasticity			

SH3BP1 (3BP-1)	Growth cone collapse	Sema3E/PlexinD1		

BARGIN	ROS diminishing		Alzheimer's disease	

CrossGAP (CrGAP/Vilse)	Axon guidance and spine morphogenesis	Slit/Robo		Cdc42

MgcRacGAP (RacGAP1/Cyk4)	Neuronal migration			
